# Genome Sequence of *Streptomyces* sp. Strain RTd22, an Endophyte of the Mexican Sunflower

**DOI:** 10.1128/genomeA.00693-16

**Published:** 2016-07-21

**Authors:** Fernanda O. Chagas, Antonio C. Ruzzini, Larissa V. Bacha, Markyian Samborskyy, Raphael Conti, Rita C. Pessotti, Luciana G. de Oliveira, Jon Clardy, Monica T. Pupo

**Affiliations:** a School of Pharmaceutical Sciences of Ribeirão Preto, University of São Paulo, Ribeirão Preto, São Paulo, Brazil; b Department of Biological Chemistry and Molecular Pharmacology, Harvard Medical School, Boston, Massachusetts, USA; c Department of Biochemistry, University of Cambridge, Cambridge, England; d Department of Organic Chemistry, University of Campinas, Campinas, São Paulo, Brazil

## Abstract

We report here the complete genome sequence of *Streptomyces* sp. strain RTd22, an endophytic actinobacterium that was isolated from the roots of the Mexican sunflower *Tithonia diversifolia*. The bacterium’s 11.1-Mb linear chromosome is predicted to encode a large number of unknown natural products.

## GENOME ANNOUNCEMENT

Endophytic bacteria, in particular those in the genus *Streptomyces*, are potential reservoirs of new antibiotic and chemotherapeutic compounds. As part of a larger research program aimed at discovering biologically active small molecules from bacterial endophytes, we isolated *Streptomyces* sp. strain RTd22 from the Mexican sunflower, *Tithonia diversifolia* (*Asteraceae*). The plants were grown and harvested in Ribeirão Preto, São Paulo, Brazil, and the isolation and identification of endophytic bacteria were performed as previously described (1).

A draft genome sequence of *Streptomyces* sp. RTd22 was generated from paired-end libraries constructed with the Nextera library preparation kit. The sequencing (2 × 250-bp [300 cycle kit]) was performed using V2 Illumina sequencing chemistry run on a MiSeq instrument and produced 7.8 million reads. After filtering and adapter and quality trimming, the reads were assembled using the Lasergene SeqMan NGen version 3.1 (DNAStar) assembler program, converted to Consed-compatible ACE format, and checked using Consed (2, 3). The draft assembly consisted of 199 contigs (made into 97 scaffolds), with an average coverage of 70×. The 199 contigs comprise 11,180,448 bp, with a G+C content of 71.3%. Annotation was carried out using a customized pipeline based on FgenesB, operating in the *ab initio* mode, and the results were edited using Artemis (4). Genome annotation using RAST (5) predicted 9,577 coding sequences, including 93 RNA genes.

To overcome the assembly issues that are commonly associated with short-read data from high-G+C organisms, we resequenced RTd22 using PacBio single-molecule real-time (SMRT) sequencing technology (6). Specifically, an insert library of an 11.5 kb was prepared, and sequence data were generated from 4 SMRT cells run on a Pacific Biosciences RSII instrument using P6-C4 chemistry. *De novo* assembly was performed using the Hierarchical Genome Assembly Process (HGAP) (7), and after manual curation to remove low-quality bases, a single contiguous sequence of 11,142,275 bp with an average G+C content of 71.3% was produced. The RAST annotation server allowed the identification of 9,667 predicted protein-coding genes, including 88 RNA genes. Analysis of the 11.1-Mb RTd22 chromosome using antiSMASH (8) predicted ~40 biosynthetic gene clusters (BGCs) for secondary metabolite production. Among the 40 predicted BGCs, very few can be confidently assigned to a known natural product based on gene and nucleotide conservation. One exception is a BGC that is predicted to encode a himastatin-like molecule (9). More generally, the chromosome appears to be enriched in terpene-related genes with other abundant classes of BGCs, including nonribosomal peptides and several polyketide clusters that encode polyene macrolides. Altogether, genome sequencing has revealed that the endophyte *Streptomyces* sp. RTd22 encodes a large chemical reservoir that merits further studies.

### Nucleotide sequence accession numbers.

The whole-genome sequence has been deposited at DDBJ/ENA/GenBank under the accession numbers LXIC00000000 (Illumina) and CP015726 (PacBio). The version described in this paper is the first version.
